# Augmented Reality‐Guided Frozen Section Analysis: Bringing the Pathologist From the Laboratory to the Operating Room

**DOI:** 10.1002/oto2.70002

**Published:** 2024-08-28

**Authors:** Marina Aweeda, Liyu Huang, Alexander N. Perez, Kim A. Ely, Mitra Mehrad, James S. Lewis, Michael C. Topf

**Affiliations:** ^1^ Department of Otolaryngology–Head and Neck Surgery Vanderbilt University Medical Center Nashville Tennessee USA; ^2^ Department of Pathology Microbiology and Immunology, Vanderbilt University Medical Center Nashville Tennessee USA; ^3^ Department of Laboratory Medicine and Pathology Mayo Clinic Phoenix Arizona USA; ^4^ Department of Biomedical Engineering Vanderbilt University Nashville Tennessee USA

**Keywords:** augmented reality, frozen section analysis, head and neck cancer, head and neck surgery, intraoperative communication

## Abstract

Due to the anatomic complexity of the head and neck and variable proximity between laboratory and operating room (OR), effective communication during frozen section analysis (FSA) between surgeons and pathologists is challenging. This proof‐of‐concept study investigates an augmented reality (AR) protocol that allows pathologists to virtually join the OR from the laboratory. Head and neck cancer specimens were scanned ex vivo using a 3‐dimensional scanner and uploaded into an AR platform. Eight head and neck specimens were discussed by surgeons and pathologists in an AR environment. AR‐guided intraoperative consultation was used for specimen orientation and discussion of FSA margin sampling sites. One patient had positive initial margins on FSA and was re‐resected to negative final margins. AR‐guided FSA is possible and allows pathologists to join the operating from any location for intraoperative discussion.

In head and neck cancer surgery, intraoperative frozen section analysis (FSA) is the gold standard for margin assessment, with virtually all head and neck surgeons using FSA.^1^ Due to the anatomic complexity of the head and neck, effective communication during FSA between surgeons and pathologists is challenging. Specimen orientation with marking sutures can help, but the ideal communication handoff is an in‐person conversation. This requires either the surgeon to leave the operating room (OR), or the pathologist to leave the laboratory. However, this is time‐consuming and may not be feasible based on location.

Augmented reality (AR) has emerged as an innovative tool in medicine. AR overlays digital content onto the real world, allowing users to interact simultaneously with physical and virtual elements. To address the challenges of intraoperative communication between surgeon and pathologist, we created a novel AR protocol for the communication of FSA results. Through a video livestream from the surgeon's AR headset, the pathologist can join the OR virtually.

## Methods

### Three‐dimensional (3D) Scanning and Specimen Mapping Technique

This study was approved by the Vanderbilt University Medical Center Institutional Review Board (IRB #221733). Using our previously established 3D scanning and specimen mapping protocols,^2^, ^3^ a 3D scanner is used to capture the surface topography of fresh ex vivo specimens. Using computer‐aided design software, a research team member annotates the model to mark the locations of inking and margin sampling sites.

### AR Visualization Workflow

**Figure 1:**
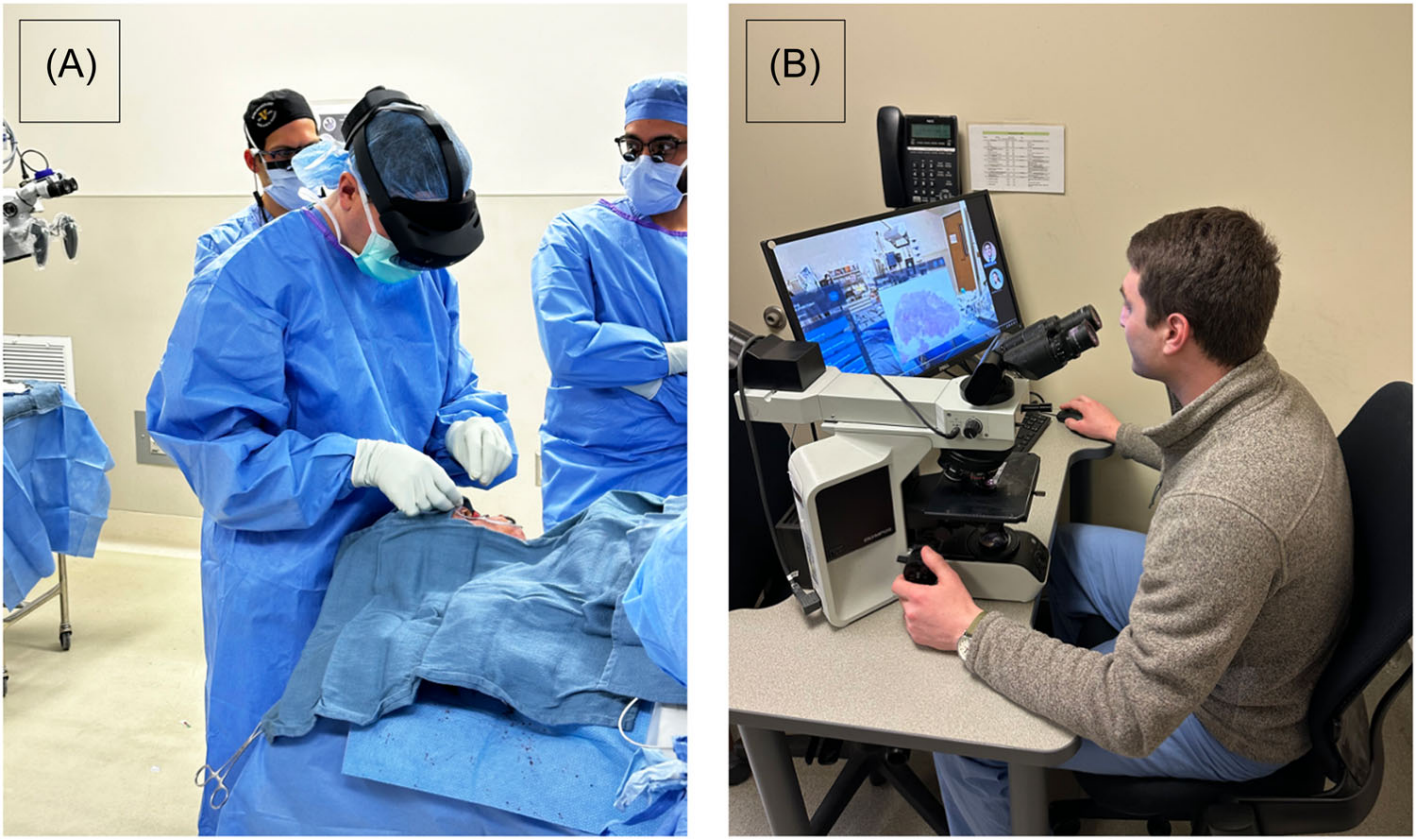
(A) Augmented reality headset is placed on the surgeon's head intraoperatively and secured. (B) Pathologist virtually joining the operating room through a teams meeting and observing the surgeon's perspective through the HoloLens 2.

**Figure 2:**
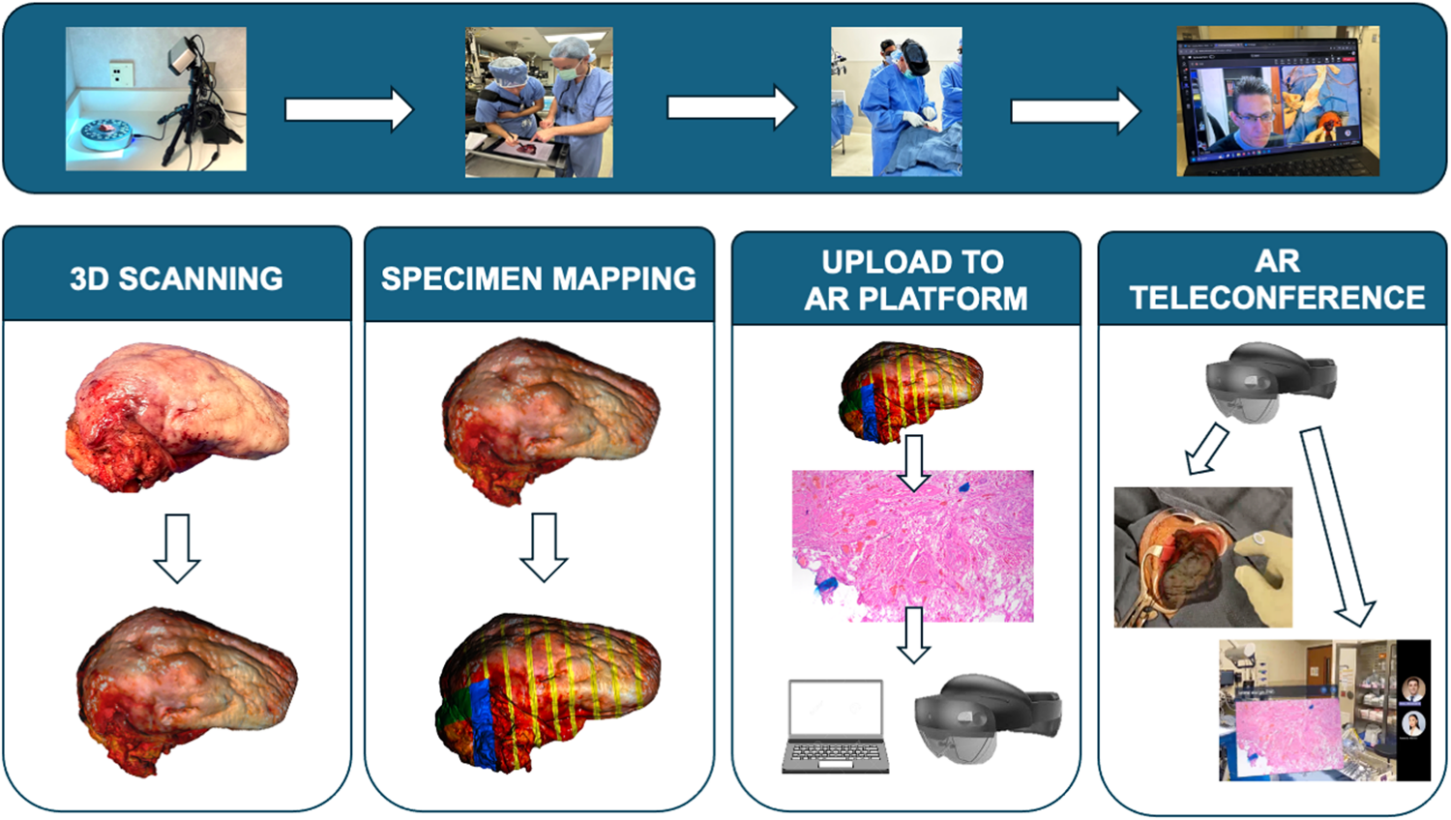
Augmented reality (AR) protocol workflow. 3D, 3‐dimensional.

The 3D model and 2‐dimensional (2D) images of the microscopic slide analysis are then uploaded into an AR platform (VSI HoloMedicine, ApoQlar). A dedicated member of the research team completes the 3D scanning, annotation, and patient profile setup. In the OR, the research team member secures the HoloLens 2 AR headset (Microsoft Corporation) onto the surgeon's head (Figure 1A). Using hand gestures and voice commands, the surgeon accesses the AR platform, displaying the uploaded digital information into the surgical field. A teleconference is initiated on the HoloLens 2 headset and the pathologist joins from the laboratory (Figure 1B). A summary figure detailing the AR protocol is shown in Figure 2.

## Results

From December 2023 to March 2024, 8 head and neck specimens were discussed intraoperatively using AR. Detailed information about the cases is available in Table 1.

**Table 1:** Case Characteristics

Case no.	Surgical procedure	Histology	Frozen section margin analysis	Turn‐around time, min	AR‐guided intraoperative consultation	Final margin status
1	Hemiglossectomy	SCC	NEGATIVE Anterior tongue (−) 7 mm from margin Lateral tongue (−)	18	Discussion of specimen orientation/margin sampling sites using 3D hologram.	NEGATIVE Closest margin: lateral, 6 mm.
2	Floor of mouth excision	SCC	NEGATIVE Ventral tongue (−): focal severe dysplasia Dorsal tongue (−): focal severe dysplasia Anterior tongue (−) Mandibular alveolar ridge (−)	36	Discussion of specimen orientation/margin sampling sites using 3D hologram.	NEGATIVE All resection margins negative for malignancy.
3	Floor of mouth excision	SCC	POSITIVE Anterior mucosal (+): papillary SCC at margin Lateral mandibular alveolar ridge (−)	26	Discussion of specimen orientation/margin sampling sites using 3D hologram.	NEGATIVE Anterior re‐resection negative for malignancy.
4	Upper lip resection, partial rhinectomy	Basosquamous carcinoma	NEGATIVE Nasal septal (−) Nasal floor (−) Upper lip and nose (−)	37	Discussion of 2 perpendicular and 1 shave margin sites. Displayed 3D mapped specimen. Displayed 2D H&E image of closest margin (upper lip).	POSITIVE Focally positive along superomedial aspect adjacent to the nares/inferior nose.
5	Partial rhinectomy, medial maxillectomy	SCC	NEGATIVE L cheek (−) L upper lip (−) L columella (−) L nasal mucosal (−)	34	Discussion of 4 perpendicular margin sites using 3D mapped specimen. Displayed 2D H&E image of L cheek margin and discussed no further need for re‐excision.	NEGATIVE Closest margin: anterior, 4 mm
6	Frontal scalp resection	Pleomorphic dermal sarcoma	NEGATIVE R medial (−) L lateral (−) Anterior (−) Posterior (−)	36	Discussion of tumor biopsy. Discussion of 4 perpendicular margin sites using 3D mapped specimen. Displayed 2D H&E image of L lateral margin, which had evidence of actinic changes.	NEGATIVE All resection margins negative for sarcoma.
7	Wide local excision of preauricular lesion	Atypical fibroxanthoma	NEGATIVE Anterior ear (−) Helical root (−) Ear canal (−) Preauricular deep (−)	32	Discussion of tumor biopsy. Discussion of 4 perpendicular margin sites using 3D mapped specimen. Displayed 2D H&E image of helical root margin, which was negative for malignancy and had evidence of solar elastoses.	NEGATIVE All resection margins negative for malignancy.
8	Subtotal glossectomy	SCC	NEGATIVE Lateral (−) Posterior (−)	31	Discussion of tumor biopsy. Discussion of 2 perpendicular margin sites using 3D mapped specimen. Displayed 2D H&E image of lateral margin, which was negative for malignancy.	NEGATIVE Closest margin: lateral, 4 mm

Abbreviations: 2D, 2‐dimensional; 3D, 3‐dimensional; AR, augmented reality; H&E, hematoxylin and eosin; L, left, R, right; SCC, squamous cell carcinoma.

### Pathology Results

Six cases (75%) had negative initial and final margins. One patient (12.5%) had positive initial margins on FSA and was re‐resected to negative final margins. One patient (12.5%) had a positive final margin due to sampling error. In this case, intraoperative FSA margins of the septum, nasal floor, and upper lip via a specimen‐based approach were negative. However, the final margin was positive in a separate, nonfrozen sampled region.

### AR‐Guided Intraoperative Consultation

AR‐guided intraoperative consultation was used for the discussion of specimen orientation and FSA margin sampling sites in all patients. 2D hematoxylin and eosin (H&E) images of margin sampling sites taken for FSA were displayed and reviewed in 5 patients, with preoperative biopsy findings discussed in 3 patients. Mean turnaround time (defined as time between the laboratory receiving the specimen to result reporting via AR teleconference) was 31 minutes (range: 18‐31).

### Illustrative Case

A 78‐year‐old male presented with a history of squamous cell carcinoma (SCC) of the base of tongue treated with chemoradiotherapy in 2013 presented with a new SCC of the right oral tongue and underwent right subtotal glossectomy (Case #8). The surgical specimen was 3D scanned and a specimen‐based approach to FSA was performed per standard‐of‐care. Perpendicular margins were taken from the posterior and lateral mucosal margins.

**Figure 3:**
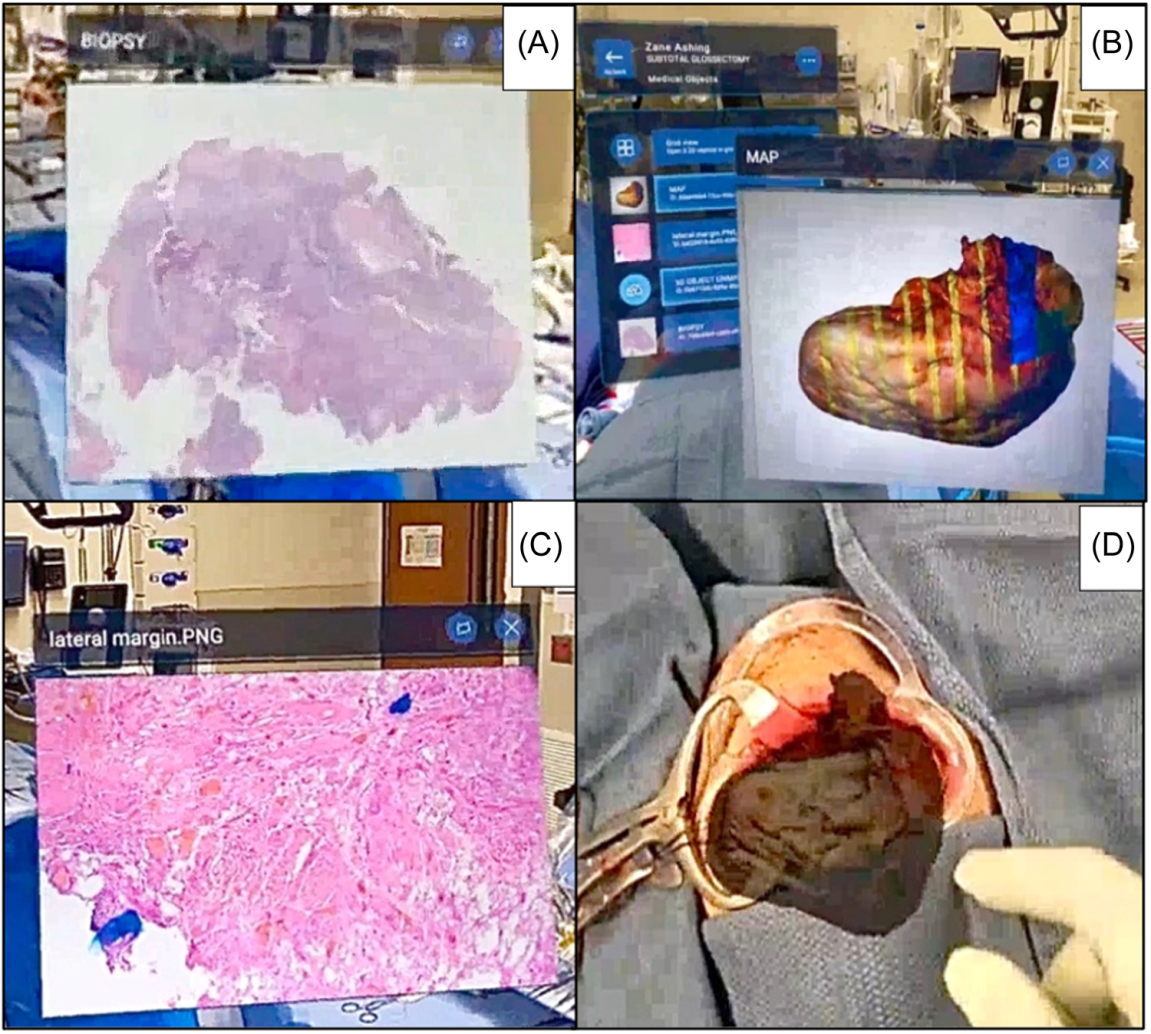
(A) Two‐dimensional (2D) image of preoperative biopsy; (B) margin sites for frozen section analysis denoted on virtually annotated 3‐dimensional (3D) specimen; (C) 2D image of lateral mucosal margin hematoxylin and eosin slide; (D) 3D model oriented in resection bed.

Using the AR platform, the surgeon displayed the 3D model of the resection, reorienting it into the resection bed to demonstrate the location of margin sampling sites. In addition, a 2D image of the H&E section from the lateral margin was reviewed together in the AR environment. This margin showed invasive SCC 4 mm from the lateral mucosal margin (Figure 3). The surgeon and pathologist discussed the close margin and decided re‐resection was not necessary (Supplemental Video S1, available online).

## Discussion

This proof‐of‐concept study demonstrates a novel use of AR to virtually bring the pathologist from the laboratory into the OR for an interactive discussion of specimen orientation, sites of margin sampling, and microscopic results. In 2024, the standard of care for surgeons and pathologists to communicate FSA results is a telephone call without any visual aid. Studies have shown that when a positive or close margin is identified, surgeons have difficulty relocating the anatomic site and resecting additional tissue.^4^ Challenges in margin relocation likely contribute to the fact that re‐resection to negative margins in head and neck malignancies fails to significantly improve oncologic outcomes.^5^ We argue that in 2024 such critical information should be communicated with visual aid.

Although we present a specific use of AR for FSA, we envision that AR‐guided intraoperative consultation could be implemented on a broader scale across a health care system. This platform could be purchased with AR headsets to facilitate intraoperative consultation in a secure, Health Insurance Portability and Accountability Act‐compliant fashion. This would allow for communication not only between the surgeon and the pathologist, but also could be leveraged for surgeon‐surgeon intraoperative consultation.

We acknowledge that this proof‐of‐concept study represents a small sample and requires further investigation. Most importantly, like any novel technology implemented in the OR, we ultimately need to demonstrate value for this approach. Moving forward, we will perform a prospective, survey‐based feasibility study with a larger sample size to assess the qualitative improvement in surgeon‐pathologist communication.

## Conclusion

AR‐guided FSA is feasible and allows pathologists to join the OR virtually from the pathology laboratory for intraoperative discussion of specimen orientation, location of margin sampling, and communication of results.

## Author Contributions

**Marina Aweeda**, design, conduct, analysis, manuscript preparation; **Liyu Huang**, conduct, analysis; **Alexander N. Perez**, conduct, analysis, manuscript preparation; **Kim A. Ely**, manuscript preparation; **Mitra Mehrad**, manuscript preparation; **James S. Lewis Jr**, design, conduct, manuscript preparation; **Michael C. Topf**, design, conduct, analysis, manuscript preparation.

## Disclosures

### Competing interests

No conflicts of interest to disclose.

## Funding source

This work was supported by an AHNS/AAO‐HNSF Young Investigator Combined Award; Vanderbilt Clinical Oncology Research Career Development Program (K12 NCI 2K12CA090625‐22A1); and Vanderbilt‐Ingram Cancer Center Support Grant (P30CA068485).

## Supporting information


**Video 1**.

## Data Availability

The authors confirm that the data supporting the findings of this study are available within the article and its supplementary materials and are available from the corresponding author, M.C.T., upon reasonable request.
